# Special Issue “Posture, Balance, and Gait: Assessment Techniques and Rehabilitation Strategies”

**DOI:** 10.3390/jfmk10040457

**Published:** 2025-11-21

**Authors:** Vasiliki Sakellari, George Gioftsos

**Affiliations:** Physiotherapy Department, Faculty of Health and Care Sciences, University of West Attica, Agiou Spidonos 28, Egaleo, 12243 Athens, Greece; gioftsos@uniwa.gr

## 1. Editorial Introduction

Posture, balance, and gait are central determinants of human movement, independence, and quality of life. Their integrity depends on the coordinated interaction of musculoskeletal, sensory, and neural systems. Impairments—whether due to aging, neurological or musculoskeletal conditions, or environmental constraints—can substantially increase the risk of falls, disability, and reduced autonomy.

This Special Issue, “Posture, Balance, and Gait: Assessment Techniques and Rehabilitation Strategies”, https://www.mdpi.com/journal/jfmk/special_issues/76CZ2L40OD (accessed on 13 November 2025), presents a curated collection of studies spanning foundational science, methodological innovations, therapeutic strategies, and applied population research. Together, these contributions represent a continuum from theory to clinical application, enhancing our understanding of human movement and guiding evidence-based rehabilitation practice.

Foundational research continues to refine our knowledge of the physiological, biomechanical, and sensory–motor mechanisms that underpin posture, balance, and musculoskeletal function. Spatial attention, for instance, operates through both goal-directed and stimulus-driven processes within the fronto-parietal cortex, enhancing multisensory processing at attended locations regardless of modality [1]. Exercise-based interventions have been shown to improve postural alignment and physical capacities, though their long-term benefits often require continuous training [2]. Conversely, sagittal spinal curves have not demonstrated strong associations with health outcomes such as spinal pain [3]. Somatosensory inputs play a pivotal role in postural control by triggering and scaling automatic postural responses [4], while the cerebellar nuclei integrate diverse sensorimotor signals to support the execution and adaptation of dexterous limb movements—processes disrupted in cerebellar dysfunction [5].

Epidemiological data emphasize the importance of accurate and objective assessment methods: in 2018, more than one in four older adults reported at least one fall, and over 10% sustained a fall-related injury [6]. These findings underline the need for precise tools that move beyond subjective observation. Wearable technologies, such as accelerometers, activity monitors, and pressure sensors, now provide noninvasive, real-time evaluations of gait parameters including cadence, stride length, gait cycle duration, and symmetry [7]. Sensor-based and motion analysis systems further enhance the precision of clinical assessment, supporting data-driven and individualized rehabilitation planning.

Progress in assessment has been matched by advances in therapeutic interventions. Wearable sensors enable clinicians to monitor gait abnormalities over time- and design-personalized rehabilitation strategies [7]. Augmented feedback derived from sensor-based gait reports offers a valuable complement to traditional assessments, improving treatment planning and outcomes in stroke rehabilitation [8].

Population-focused research provides additional clinical relevance. Osteoarthritis, for example, is strongly associated with limitations in activities of daily living among older adults. Hip or knee osteoarthritis significantly affects mobility, self-care, and domestic activities, with some evidence suggesting stronger effects in men for specific mobility-related tasks [9]. As global mobility limitations rise, such insights underscore the need for targeted, population-specific rehabilitation approaches.

The contributions in this Special Issue can be conceptually grouped into four thematic areas that reflect the translational pathway of rehabilitation research:

1. Foundational and Conceptual Frameworks—studies elucidating the physiological, biomechanical, and sensory–motor bases of posture, balance, and movement.
2. Clinical Assessment Methods—research developing and validating objective measurement tools and predictive models for postural control and gait performance.
3. Rehabilitation Strategies and Interventions—evidence-based approaches ranging from sensory stimulation and exercise to robotic and sensor-assisted therapies.
4. Population-Specific and Applied Research—investigations focusing on distinct cohorts such as athletes, older adults, or patients undergoing surgery.

Collectively, the studies presented in this Special Issue advance both scientific knowledge and clinical practice, offering innovative perspectives on how posture, balance, and gait can be preserved, improved, and optimized across the lifespan.

## 2. Highlights of Contributions

### 2.1. Foundational and Conceptual Frameworks (Contribution 1 and 2)

Two contributions, Lerida-Ponce et al. (Contribution 1) and Akao et al. (Contribution 2), provide fundamental insights into the physiological, biomechanical, and sensory–motor mechanisms underlying posture, balance, and musculoskeletal function. Lerida-Ponce et al. focus on the interplay between visual demands and cervical muscle activation, revealing how ocular input integrates with motor control and proprioception. Akao et al. demonstrate how spinal–pelvic alignment and intersegmental interactions shape muscle length and postural control. Together, they establish a conceptual framework that informs both research and clinical approaches to assessment and rehabilitation.

Lerida-Ponce et al. (2025, Contribution 1) conducted a systematic review investigating the links between visual accommodation, cervical muscle activity, and related symptomatology, analyzing seven experimental studies with 308 participants. The findings indicated that accommodative stress consistently increased trapezius electromyographic activity, suggesting a direct connection between ocular effort and cervical muscle activation. This work highlights a functional and anatomical coupling between the visual and cervical systems, expanding the conceptual framework of posture by integrating visual demands with proprioceptive and motor mechanisms. Clinically, it supports interdisciplinary interventions, including optometric training, ergonomic adjustments, and postural re-education to mitigate cervical strain associated with visual fatigue.

Complementing this perspective, Akao et al. (2025, Contribution 2) examined spinal–pelvic alignment and its relationship to muscle shortening in 41 healthy young men, using Shapley Additive Explanation (SHAP) analysis to assess individual and interactive effects of thoracic kyphosis, lumbar lordosis, and anterior pelvic tilt on six key muscles. Results showed that hip-related muscles—iliopsoas, rectus femoris, gluteus maximus, and hamstrings—were strongly influenced by both individual alignments and intersegmental interactions, particularly the thoracic–lumbar interplay. In contrast, back extensors and abdominals were minimally affected. These findings emphasize the need to consider segmental spinal interactions in postural assessment and muscle flexibility interventions.

### 2.2. Clinical Assessment Methods (Contribution 3, 4, 5, 6 and 7)

A second group of contributions in this Special Issue (Contribution 3, 4, 5, 6 and 7) addresses the evaluation of postural control, gait, and musculoskeletal function, emphasizing objective measurement, psychometric validation, and clinically meaningful interpretation.

Mirando et al. (2025, Contribution 3) investigated the distribution of plantar pressures during quiet stance and gait in 60 healthy adults using a baropodometric platform. The study assessed pressures across ten regions of the foot, revealing that although gait increased overall plantar pressures, the spatial distribution patterns closely resembled those observed during quiet stance. Significant positive correlations between static and dynamic measurements suggested that plantar pressure during gait could, to some extent, be estimated from quiet stance data. Clinically, this provides a practical approach for assessing foot loading, guiding orthotic prescription, and informing postural and gait rehabilitation. By integrating static and dynamic assessments, clinicians can efficiently evaluate foot biomechanics even in settings with limited sensorized equipment, supporting interventions that reduce injury risk and optimize functional performance.

Polo-Ferrero et al. (2025, Contribution 4) focused on fall risk assessment in older adults by evaluating the predictive capacity of the 3-Meter Backward Walk Test (3m-BWT) in 483 community-dwelling participants aged 60–96 years, including 101 individuals with a prior history of falls. Results indicated that slower 3m-BWT performance was significantly associated with previous falls and moderately correlated with established functional assessments, including the Short Physical Performance Battery, Five-Repetition Sit-to-Stand, gait speed, Timed Up-and-Go, and Four-Square Step Test. Optimal cut-off points were proposed to stratify risk, balancing sensitivity and specificity. Although no single test can fully capture the multifactorial nature of falls, the 3m-BWT offers a rapid, easily administered, and sensitive tool to complement comprehensive geriatric evaluations, supporting early identification of individuals at risk and informing targeted preventive interventions.

Gianzina et al. (2025, Contribution 5) extended the application of objective clinical assessment to a clinical population, examining gait impairments in individuals awaiting total knee arthroplasty compared with healthy age-matched controls using wearable inertial sensors. The study assessed 17 gait parameters, including stride duration, cadence, symmetry indices, pelvic tilt, rotation, and obliquity, and propulsion indices for both legs. Patients awaiting TKA demonstrated significantly longer gait cycles, reduced cadence, lower symmetry indices, and decreased propulsion compared with controls, highlighting compensatory gait strategies adopted to reduce knee joint load. The findings emphasize the potential of wearable sensors to provide objective, high-resolution biomechanical data for prehabilitation, functional monitoring, and tailored rehabilitation programs aimed at improving gait performance prior to surgery. This approach also underscores the importance of assessing multiple gait dimensions rather than relying solely on global measures, enhancing sensitivity to subtle functional impairments.

Matsuda et al. (2025, Contribution 6) used two small motion sensors placed on the shank and lower back to estimate knee loading during walking at different step rates. The method showed strong agreement with standard 3D motion analysis, particularly at normal or faster walking speeds, suggesting a simple and practical way to assess knee biomechanics in clinical settings.

Coetzee et al. (2025, Contribution 7) employed a preoperative clinical phenotyping approach in patients with end-stage knee osteoarthritis awaiting total knee replacement in South Africa. Using exploratory factor analysis on a combination of demographic, functional, physical, and patient-reported variables, three distinct phenotypes were identified: (1) gait and weight—characterized by poor gait mechanics, obesity, and low self-efficacy; (2) central pain—encompassing central sensitization, depressive symptoms, and reduced functional performance; and (3) functional factors—reflecting muscular weakness and sedentary behavior. These phenotypes illustrate the heterogeneity of clinical presentations in end-stage knee osteoarthritis and highlight the need for tailored, multidisciplinary preoperative interventions addressing gait, strength, pain management, psychosocial support, and behavioral change strategies. By stratifying patients according to phenotypic characteristics, clinicians can optimize rehabilitation and enhance post-surgical outcomes.

### 2.3. Rehabilitation Strategies and Interventions (Contributions 8, 9, and 10)

This group of contributions is classified under “Rehabilitation Strategies and Interventions”. Each test structured therapeutic approaches designed to improve balance, gait, and motor recovery in diverse populations. These works evaluate the efficacy of active interventions—ranging from sensory stimulation protocols to treadmill-based and robotic therapies—that aim to enhance postural control, functional mobility, and independence. Taken together, these three contributions reflect the breadth and innovation of contemporary rehabilitation strategies. They highlight the shift toward multimodal, evidence-based rehabilitation strategies that are tailored to both populations needs and technological possibilities. As a group, Contributions 8, 9, and 10 demonstrate how physiotherapy research is increasingly positioned at the intersection of sensory science, motor learning, and technological innovation, paving the way for more precise, effective, and accessible rehabilitation solutions.

Tanaka et al. (2025, Contribution 8) investigated the effects of a novel sensory stimulation protocol targeting the trunk and plantar surfaces in older adults. Using a randomized design, the authors demonstrated that repeated tactile stimulation significantly improved dynamic balance, as measured by center-of-pressure displacement and stability indices. These findings suggest that augmenting sensory input from key postural regions can compensate for age-related declines in proprioception and somatosensation. By activating sensory–motor integration pathways, this low-cost and non-invasive intervention offers a promising avenue for fall prevention and functional mobility support in geriatric populations.

Lepoura et al. (2025, Contribution 9) extended the focus to pediatric neurorehabilitation, evaluating Functional Partial Body Weight Support Treadmill Training (FPBWSTT) in children with ataxia. The study found that structured treadmill-based training improved gait symmetry, walking speed, and dynamic balance, while also reducing ataxic movement patterns. Importantly, the intervention was well tolerated by children, underscoring its clinical feasibility and potential as a complementary therapy in managing balance and mobility deficits associated with pediatric ataxia. By integrating partial weight support with functional, repetitive locomotor practice, FPBWSTT highlights the therapeutic value of task-specific, intensive training paradigms for children with neurological disorders.

Bonanno et al. (2025, Contribution 10) explored the application of robotic-assisted gait training compared with conventional physiotherapy in individuals with chronic stroke. Their randomized controlled trial demonstrated that robotic training resulted in superior improvements in gait parameters, balance control, and lower-limb motor function. The findings underline the ability of robotic systems to deliver high-intensity, repetitive, and precisely controlled locomotor practice, which may surpass the dosage and consistency achievable in standard therapy. By harnessing advanced technology, robotic gait training exemplifies a forward-looking approach that complements therapist-led rehabilitation while expanding therapeutic possibilities for individuals with persistent motor impairments.

### 2.4. Population-Specific and Applied Research (Contribution 11)

Classified under “Population-Specific and Applied Research”, this study shows how examining surfers yields insights not captured in broader populations. By addressing the unique demands of aquatic sports, it demonstrates that targeted activity enhances postural stability and flexibility. Beyond sport performance, these results have translational value for rehabilitation and fall-prevention, illustrating how population-centered research can inform applied health and performance practices.

De Castro-Maqueda et al. (2025, Contribution 11) investigated balance, flexibility, and self-esteem in surfers compared to non-surfers and physically active controls, employing established clinical measures such as the SEBT, Flamenco Test, Sit-and-Reach, and Rosenberg Scale. The study found that surfers demonstrated superior static and dynamic balance, suggesting sport-specific adaptations that surpass the benefits of general physical activity, while no differences emerged in self-esteem.

## 3. Translational Significance

The collective contributions of this Special Issue underscore the relevance of posture, balance, and gait research for clinical translation. For example, Lerida-Ponce et al. (2025, Contribution 1) identified physiological links between visual accommodation and cervical muscle activity, suggesting that visual demand may influence musculoskeletal tone and pain pathways, with implications for patient management in cervical disorders. Similarly, Mirando et al. (2025, Contribution 3) demonstrated that plantar pressure distribution during gait can be reliably estimated from quiet stance in healthy adults, a finding that may streamline clinical screening protocols and broaden assessment strategies beyond laboratory contexts. Translational value is also evident in the applied studies: Tanaka et al. (2025, Contribution 8) showed that vibratory sensory stimulation of the trunk and sole significantly improves balance and reduces fall risk in older adults, while Lepoura et al. (2025, Contribution 9) provided robust evidence that functional partial bodyweight support treadmill training enhances motor performance and daily mobility in children with ataxia. Complementary to these approaches, Polo-Ferrero et al. (2025, Contribution 4) validated the 3-Meter Backward Walk Test as a promising, though not stand-alone, predictor of fall risk in older adults, underscoring the need for multidimensional screening. Together, these findings illustrate how biomechanical insights, population-specific adaptations, and innovative rehabilitation techniques can be translated into practical strategies for prevention, assessment, and intervention.

## 4. Future Directions

While the studies in this Special Issue advance both theoretical understanding and clinical application, they also identify key knowledge gaps that outline a forward-looking research agenda. Akao et al. (2025, Contribution 2) underscored the importance of analyzing the interactive effects of thoracic, lumbar, and pelvic alignment on muscle shortening, calling for future investigations that incorporate strength measures and frontal-plane parameters into comprehensive, multidimensional models. Likewise, Gianzina et al. (2025, Contribution 5) reported notable gait impairments among individuals awaiting total knee arthroplasty and emphasized the need for prehabilitation research—particularly interventions integrating strengthening and wearable-sensor feedback—to enhance surgical outcomes. Bonanno et al. (2025, Contribution 10) demonstrated that end-effector robotic gait training may improve motor coordination even in chronic stroke, yet they highlighted the necessity for long-term and cost-effectiveness studies to establish sustainability and clinical value. De Castro-Maqueda et al. (2025, Contribution 11) introduced an innovative perspective by showing that surfing can enhance postural stability, suggesting potential therapeutic applications for balance disorders if validated within controlled rehabilitation settings. Finally, Coetzee et al. (2025, Contribution 7) advanced clinical phenotyping in end-stage knee osteoarthritis, identifying patient subgroups based on gait characteristics, body weight, central sensitization, and functional impairment—thereby supporting the development of phenotype-specific rehabilitation strategies.

Taken together, these directions trace a translational pathway that integrates biomechanical, sensory, technological, and population-focused perspectives, moving toward more personalized and effective rehabilitation paradigms.

## 5. Conclusions

Taken together, the contributions in this Special Issue illustrate how conceptual advances, methodological innovations, targeted interventions, and applied research collectively shape a coherent framework for translating posture, balance, and gait science into effective clinical and real-world applications. Progress in this field clearly depends on bridging foundational neurophysiological and biomechanical mechanisms with validated assessment tools, developing and testing innovative rehabilitation strategies, and addressing population-specific needs.

This growing body of work reflects a clear shift toward individualized, evidence-based care—emphasizing both prevention and functional recovery across diverse populations. Building on the success of this first edition, we are pleased to announce the launch of the second edition of the Special Issue, “Posture, Balance, and Gait: Assessment Techniques and Rehabilitation Strategies—2nd Edition” https://www.mdpi.com/journal/jfmk/special_issues/C731740M3W (accesssed on 13 November 2025). We warmly invite researchers and clinicians to contribute new insights, technologies, and interdisciplinary approaches that will continue to advance this dynamic and clinically meaningful area of rehabilitation science.
